# 

**Published:** 1852-04

**Authors:** 


### CONTENTS OF THE DENTAL REGISTER OF THE WEST.

APRIL, 1852.

PART I.—ORIGINAL COMMUNICATIONS.

PAGE.
Dr. Wright's Address to Graduating Class O. C. D. S., ....... 129
Report of the M. V. A. D. S. reviewed by Dr. J. Taylor, ....... 142
Dr. James Taylor's Communication to A. J. D. S. ............ 148
Association of Ohio C. D. S. ............................. 164
Congenital Absence of the Lateral Incisors, ................. 169
Dr. Baxter's Condensing Forceps, ......................... 170

PART II.—SELECTIONS.

Proceedings of the Annual Meeting of the A. S. D. S. continued, 172
Dr. Allen's Letter, ....................................... 181
Dr. Rosting, ............................................ 183

PART III.—EDITORIAL.

Dr. Allen's Improvement, ................................. 184
Dr. Hunter's Improvement, ................................ 186
A new Metalic Compound for Casts, ........................ 187
Fine Gold Fillings, ...................................... 187
Receipts for Register, ................................... 189

